# Factors and optimizations of healthcare workers' perception in alternative care facilities

**DOI:** 10.3389/fpubh.2022.891503

**Published:** 2022-07-27

**Authors:** Hao Wang, Peng Luo, Yimeng Wu, Xianqi Zeng

**Affiliations:** ^1^School of Architecture, Harbin Institute of Technology, Harbin, China; ^2^Key Laboratory of Cold Region Urban and Rural Human Settlement Environment Science and Technology, Ministry of Industry and Information Technology, Harbin Institute of Technology, Harbin, China; ^3^College of Architecture and Urban Planning, Tongji University, Shanghai, China

**Keywords:** alternative care facility (ACF), healthcare workers' perception, grounded theory, nurse-engineer partnership, active and passive strategies

## Abstract

**Background:**

Diverse measures have been carried out worldwide to establish Alternative Care Facilities (ACFs) for different ends, such as receiving, curing or isolating patients, aiming to cope with tremendous shock in the urban medical system during the early passage of the COVID-19 epidemic. Healthcare workers always felt anxious and stressed during multiple major public health emergencies in medical facilities. Some active measures to improve healthcare workers' perceptions, such as temporary training, workflow improvement, and supplementary facilities, were proved insufficient in several past public health emergencies. Therefore, this study aims to analyze the contributing factors of the healthcare workers' perceptions of the ACFs in this pandemic, which can help find an innovative path to ensure their health, well-being and work efficiency.

**Method:**

This paper conducted semi-structured in-depth interviews with the world's first batch of healthcare workers who have worked in ACFs through a qualitative study based on Grounded Theory. The healthcare workers interviewed from Heilongjiang, Shandong, Fujian, and Hubei provinces, have worked in one of the four different ACFs built in Wuhan. The results are obtained through the three-level codes and analyses of the interview recordings.

**Results:**

The factors affecting the perception of healthcare workers in ACFs during the epidemic situation can be summarized into five major categories: individual characteristics, organization management, facilities and equipment, space design, and internal environment. The five major categories affecting the composition of perception can be further divided into endogenous and exogenous factors, which jointly affect the perception of healthcare workers in ACFs. Among them, individual characteristics belong to endogenous factors, which are the primary conditions, while other categories belong to exogenous factors, which are the decisive conditions.

**Conclusion:**

This paper clarifies factors affecting the perception of healthcare workers in ACFs and analyzes the mechanism of each factor. It is posited that the passive strategies are a promising solution to protect healthcare workers' health, improve their work efficiency, and help reduce the operation stress of ACFs. We should train multidisciplinary professionals for future healthcare and enhance collaborations between healthcare workers and engineers. To sum up, this paper broadens new horizons for future research on the optimization of ACFs and finds new paths for alleviating healthcare workers' adverse perceptions of ACFs.

## Introduction

The scarcity of medical resources is ubiquitous worldwide, resulting from the large number of patients caused by the COVID-19 pandemic (1–5). Alternative Care Facilities (ACFs) are temporary facilities that can meet the emergency needs of medical treatment in public health emergencies to alleviate the burden of medical conditions of existing medical facilities (6, 7). Lam C, Waldhorn R, and others believe that there are several uses for ACFs: as overflow hospitals providing a full range of care; for limited supportive care for noncritical patients; as primary triage and rapid patient screening centers; for quarantine; etc. (8, 9). ACFs have played various roles in different countries and regions according to their medical system in this epidemic (10–15). For example, NHS Nightingale Hospital in the UK provides comprehensive care for patients (16), and Fangcang shelter hospitals in China mainly focus on isolation and provide limited supportive treatment (17–19). In general, ACFs are a common way for many countries to solve the shortage of medical facilities.

Perception is the human body's organization, identification, and interpretation of acquired information through the sensory system to present the information or environment (20). Relevant studies show that although people's perceived risk in a dangerous environment is not necessarily the same as the actual risk, the individual's perception will still affect their behavior (21, 22). Specifically, although healthcare workers are unrecognized in their nosocomial surroundings, their stress perception also impacts their health and work performances. For example, the sound and light in the hospitals will also affect the workers' stress and job satisfaction (23–26). Healthcare workers play critical roles in public health emergencies and provide emergency medical services to people in need (27–32). However, previous studies have shown that healthcare workers might have poor physical and mental health due to lack of support, increasing workload, fear of infection, and insufficient training, during public health emergencies like SARS and MERS (33–39). Moreover, there are also studies showing that healthcare workers in various countries face similar situations during the COVID-19 epidemic (40–45). And specific relevant researches on healthcare workers in ACFs show that their adverse perception may be exacerbated due to their maladjustment to the new environment, the limited medical resources and open space for activities, and the imbalance between the ratio of healthcare workers to patients (46–49).

Healthcare workers' perception of ACFs is the overall presentation of information generated in the working process through a series of their sensory systems. Traditionally, the point of view of medical staff has been measured by using questionnaires that monitor the satisfaction with the care received. However, the exclusive use of surveys to study overall health care quality has some weaknesses, including framing the protagonists' subjective experiences into rigid categories imposed by the researchers based on preconceived ideas (50). Thus, Grounded Theory constructs symbolic codes based on categories emerging from recorded qualitative data, which is quite different from the traditional scientific research model (51–53). Some practice researches understood nurses' experience with nursing consultations in the context of the Family Health Strategy and proposed a representative model with the open, axial and selective coding (54). There is also research into nurses' changing perceptions regarding the efforts in preparation for working in a COVID-19 ward in the rural Japanese context (55). Moreover, other researches explored the perception of entrepreneurship among nurses and developed a mid-range theory that explains the meaning and practices of entrepreneurship among nurses (56). The above researches fully show that the Grounded Theory method is feasible to comprehensively explain the factors affecting the perception of healthcare workers under specific conditions. Thus, to improve the adverse perception affecting healthcare workers' health, well-being and work efficiency during the epidemic, this paper clarifies the contributing factors to healthcare workers' perception of ACFs through the method of Grounded Theory, to find innovative improvement measures and alleviate their adverse perception.

## Methods

### Research method

**Figure 1:**
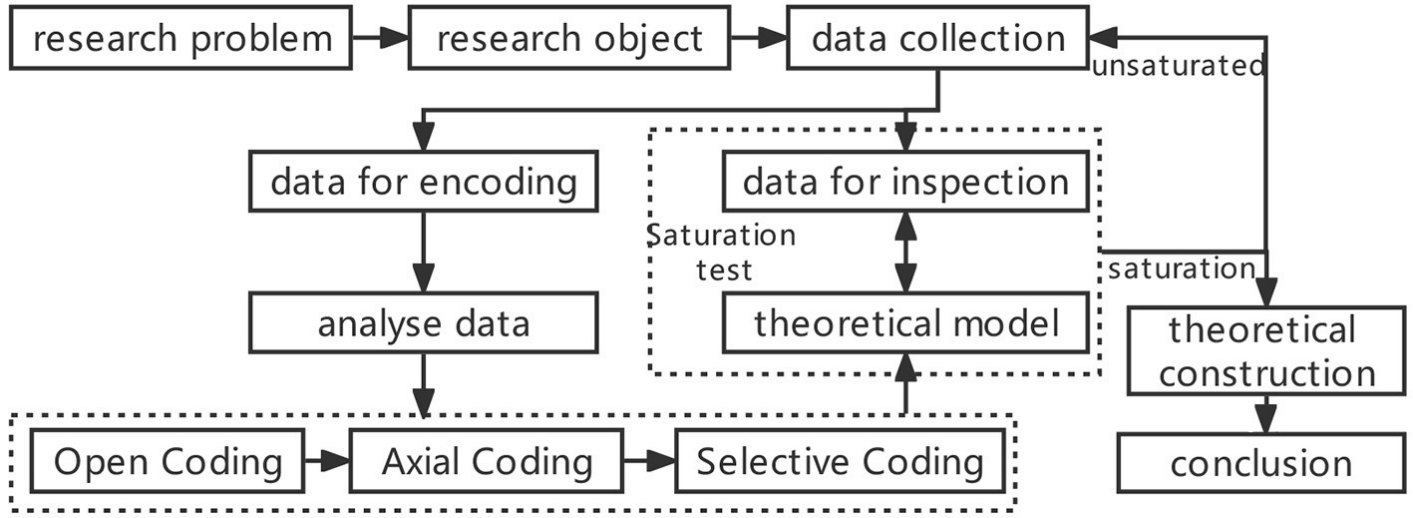
Grounded theory research process.

Grounded Theory is based on investigations and analyses by returning to the phenomenon itself and avoiding presupposition by the researchers. Categories are divided *via* concept extraction, induction, and summary in a bottom-up way based on data collected and the relationship between various categories is further explored to establish a theoretical model to solve the research questions. Specifically speaking, research processes of the Grounded Theory can be divided into four steps: research question–data collection–data analysis–theoretical construction, among which data analysis, as the core link, is usually categorized by the three-level codes, namely open coding–axial coding–selective coding (57, 58) (Figure 1).

### Participants

The selected participants were the first ones who worked in ACFs in the world during the epidemic in Wuhan, and thus there were no referential experiences for them. Hence, later policies and improvement measures did not affect their behaviors and perceptions.

The participants were eight healthcare workers who come from Hubei (2 participants), Heilongjiang (3 participants), Shandong (2 participants), and Fujian Provinces (1 participant) in China with an average age of 38.9 (SD = 6.9; min = 27; max = 50), including four men and four women. These participants included five nurses and three doctors with an average working seniority of 15.6 (SD = 9.0; min = 4; max = 30) with bachelor's degree (Table 1). To ensure the objectivity of the research results, the selection of participants in this study were from the four ACFs in Wuhan named Shipailing Fangcang shelter hospital (2 participants), Zhuankou Fangcang shelter hospital(3 participants), Guobo Fangcang shelter hospital (2 participants), Guanggu Fangcang shelter hospital (1 participant) with the same functions and ends in the same period.

**Table 1:** Demographic characteristics of respondents.

	***n***	**%**
**Gender (*****N*** **=** **8)**		
Male	4	50%
Female	4	50%
**Age (*N* = 8)**		
20–29	1	12.5%
30–39	3	37.5%
40–49	3	37.5%
50–60	1	12.5%
**Occupation (*N* = 8)**		
Nurse	5	62.5%
Doctor	3	37.5%
**Position titles (*N* = 8)**		
Associate Professor	3	37.5
Head Nurse	1	12.5%
Associate Chief of Nursing	1	12.5%
Nurse–in–charge	1	12.5%
Nurse	2	25%
**Working seniority (year) (*N* = 8)**		
0–9	1	12.5%
10–14	3	37.5%
15–19	2	25%
20–24	1	12.5%
>25	1	12.5%
**Province (*N* = 8)**		
Heilongjiang	3	37.5%
Shandong	2	25%
Fujian	1	12.5%
Hubei	2	25%
**Educational background (*N* = 8)**		
Undergraduate	8	100%

Informed consent was obtained from all participants before the interview began. Participants were informed about the goals and contents of the study, privacy, and data protection and that their participation in the study was voluntary. Biological samples were not collected.

### Data collection

This study draws up an outline for the interview as follows. There are four parts of the interview, which are not conducted in a fixed order to avoid interrupting the interviewees.

(1) *Basic information: the name of ACF, the stationed time of the healthcare workers, the number of patients, etc.;*
(2) *Work contents: the respondents' work division, organization and process, as well as the problems they encountered in ACFs, etc.;*
(3) *Perception: recognition of the respondents in different positions and at other times in ACFs from the beginning to the end;*
(4) *Improvement suggestions: the management, operation and layout design of the ideal ACFs from respondents' perspectives*.

This study has conducted interviews either online or offline because, on the one hand, interviewees are from various medical care teams in different provinces; on the other hand, it can avoid the interactions between respondents. The critical information was recorded during the 1h to 1.5h interview. Furthermore, the respondents' personal information was not mentioned so that they could tell their actual perceptions. After the interview, the interviewers analyzed the recordings.

According to the Grounded Theory, researchers will not be able to obtain new information from the research data when the research results reach saturation (51). After analyzing the interview recordings of 8 healthcare workers, the researchers found that the interview contents of the other three could not provide any new concept, and hence results are considered saturated.

## Results

### Concept and category

Open coding is a process of the label, conceptualizing and categorizing the similar or relevant information from the recordings of the interviews. This study is in accordance with the following procedures: labeling (analyze the recordings, sift essential information out, and label as “a_n_”)—conceptualization (combine similar and relevant labels, and conceptualize as “aa_n_”)–categorization (classify the conception and categorize as “A_n_”)—open coding. In all, there are 406 labels, 53 concepts, and 27 categories after the process of open coding (Table 2).

**Table 2:** Open coding process.

**a**_n_ **Label**	**aa**_n_ **Conceptualization**	**A**_n_ **Categorization**
a_1_ We got up at 8 a.m. on the 16th. A group of people from the National Health Commission trained us to wear protective clothing, prevent infection and wear masks. a_2_ I started working on the 17th, and I didn't have enough protective clothing at that time. a_3_ We stipulated six hours for each person, but at first, some people worked at least eight hours or even ten hours. a_4_ In the beginning, I was pretty unfamiliar with my work and environment. The first two groups of workers were not as smooth as expected. a_5_ I went in on the 17th in protective clothing with the high psychological pressure since the mood of rehearsal and practice in the hotel is entirely different. a_6_ We entered through the gate, and the staff had a password. a_7_ Before entering, it is a container. We must first put on our protective clothing in a sterile environment. ……	aa_1_ training before entry aa_2_ insufficient initial protective materials aa_3_ work overtime aa_4_ unfamiliarity aa_5_ tension aa_6_ the room with protective clothing is not divided aa_7_ mirrors for healthcare workers aa_8_ process of wearing protective clothing aa_9_ medical passage is equipped with a password aa_10_ large number of patients admitted internally aa_11_ a large number of patients to be cared for by each medical care provider aa_12_daily work content of medical care aa_13_ trouble caused by protective clothing aa_14_ proportional collocation, grouped action aa_15_ long walking path aa_16_ auxiliary facilities aa_17_ interaction with patients to alleviate patients' psychological problems ……	A_1_ business training A_2_ material reserve A_3_ working strength A_4_ individual mentality A_5_ medical passage A_6_ medical auxiliary facilities A_7_ working pressure A_8_ work content A_9_ walkway layout A_10_ work division A_11_ coordination and organization A_12_ peripheral medical facilities A_13_ physiological differences A_14_ previous experience A_15_ bed space A_16_ nurse station space A_17_ medical auxiliary space A_18_ patient participation A_19_ patient passage A_20_ activity space A_21_ internal ventilation A_22_ night lighting A_23_ communication A_24_ somatosensory temperature A_25_ peripheral living facilities A_26_ monitoring facilities A_27_ shared facilities

### Main category

Axial coding aims to merge correlated categories, find the links among all categories, then simplify and differentiate them. In this study, the major categories are sifted out to better specify the themes of the interview recordings by merging the minor categories together. Thus, there are five major categories after axial coding, namely “individual characteristics,” “organization and management,” “space design,” “internal environment” and “facilities and equipment” (Table 3).

**Table 3:** Main category and corresponding category.

**Number**	**AA**_n_ **Main category**	**A**_n_ **corresponding category**
1	AA_1_ Individual characteristics	A_4_ individual mentality A_13_ physiological differences A_14_ previous experience
2	AA_2_ Organization management	A_1_ business training A_2_ material reserve A_3_ working strength A_7_ working pressure A_8_ work content A_10_ work division A_11_ coordination and organization A_18_ patient participation
3	AA_3_ Space design	A_5_ medical passage A_9_ walkway layout A_15_ bed space A_16_ nurse station space A_17_ medical auxiliary space A_19_ patient passage A_20_ activity space
4	AA_4_ Internal environment	A_21_ internal ventilation A_22_ night lighting A_23_ communication A_24_ somatosensory temperature
5	AA_5_ Facilities and equipment	A_6_ medical auxiliary facilities A_12_ peripheral medical facilities A_25_ peripheral living facilities A_26_ monitoring facilities A_27_ shared facilities

### Core category

**Figure 2:**
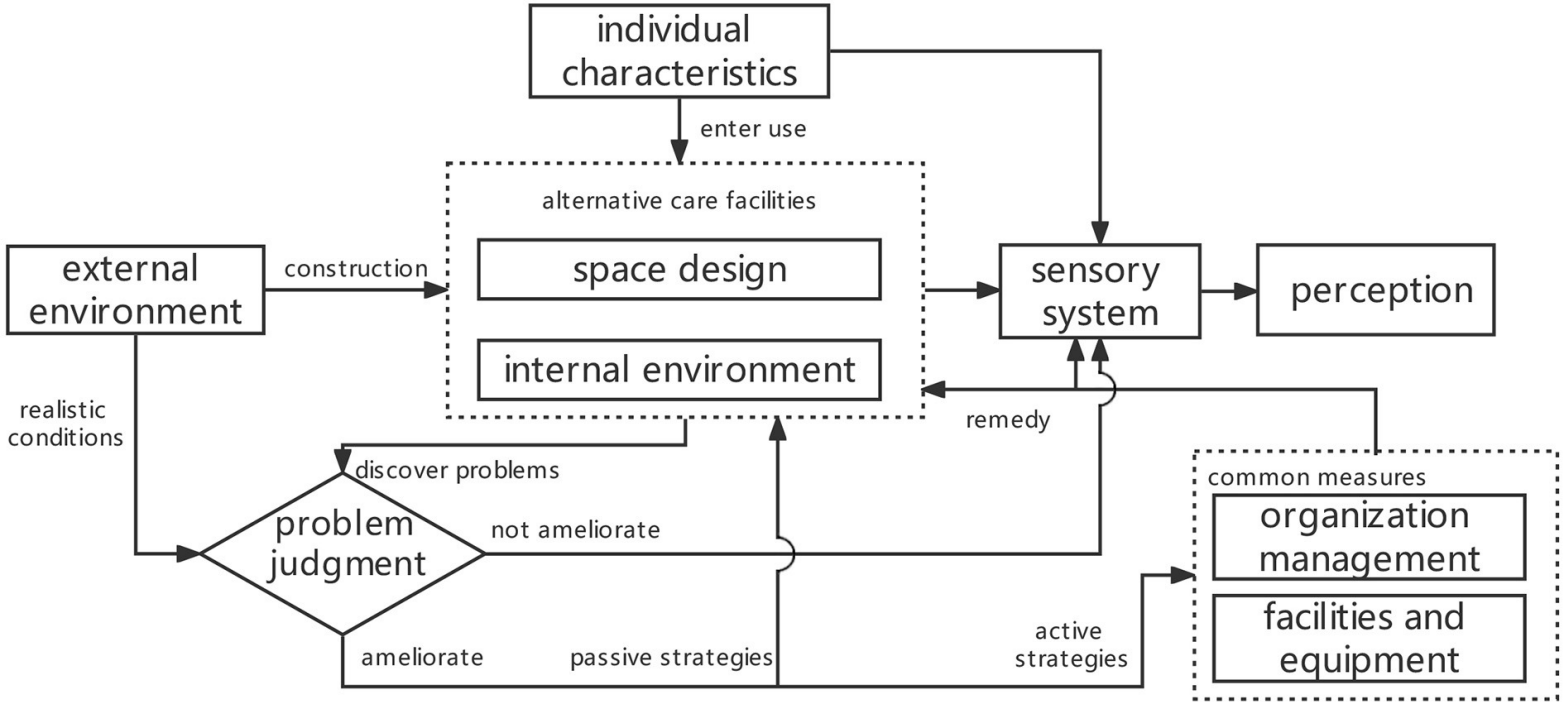
Interaction mechanism between main categories.

Selective coding aims to sift core categories from the major categories. Core categories are used to clarify the interrelation of the major ones for an integral logic to better clarify the interrelation among the major categories. It is posited that “space design” should be selected as the core category. Specifically, based on the perception of the healthcare workers in ACFs, this study takes the five major categories and other minor ones and some related conceptions into consideration, which shows that “space design” can be used to explain the correlation among the major categories. The integral logic among the five categories is as follows: because of the COVID-19 epidemic, healthcare workers with distinguishing “individual characteristics” had to work in ACFs that were not well-equipped. While the original building structures constrained the “space design” of the ACFs, the “internal environment” was relatively deficient. The inadequacies of the “space design” of the ACFs were balanced mainly through “organization management” and together with some “facilities and equipment” to improve the health, well-being and work efficiency of the healthcare workers (Figure 2).

### Relational structure

The “individual characteristics” are essential to all perceptions of the healthcare workers after clarifying the categories. While the facility and operation conditions of the ACFs were decisive factors for the final perception of healthcare workers. The “facility conditions”, i.e. space and environment of the ACFs, affect the healthcare workers' perceptional system as soon as they begin to work in the ACFs. However, the “operation conditions”, i.e. “organization management” and “facilities and equipment,” plays decisive roles in the perception of the healthcare workers. The space of ACFs is essential to healthcare workers' activities, while the environment of the buildings is rather critical to their perception. Both of them had potential impacts on the healthcare workers, although they usually seemed to be unrecognized in the space and environment (17). However, despite the limited conditions during the epidemic, some counterbalanced measures were carried out to optimize the “operation conditions” of the ACFs, aiming to improve the workers' perceptions. Primary measures were to improve management capacity and secondary ones to strengthen support facilities (Table 4).

**Table 4:** Relational structure of the main category.

**Relational structure**				**Intension**
Individual characteristics	Essential conditions			Differences in experience, gender, and stress resistance are the primary factors leading to the different perception.
Space design	Decision condition	Facility conditions	Basic problems	The design of ACFs only meets basic user needs, which is the core reason for adverse perception.
Internal environment			Core problems	The internal environment of ACFs is mainly based on safety, and the importance of perception is relatively low.
Organization management		Operation conditions	Main measures	When external conditions are limited, and it is challenging to improve the building and environment facilities, strengthening operation conditions can effectively enhance the perception, such as business training, organization, and coordination.
Facilities and equipment			Auxiliary measures	Strengthening personal protective equipment, using existing facilities , taking mobile equipment and other feasible measures can effectively improve the specific perception.

### Theoretical model

**Figure 3:**
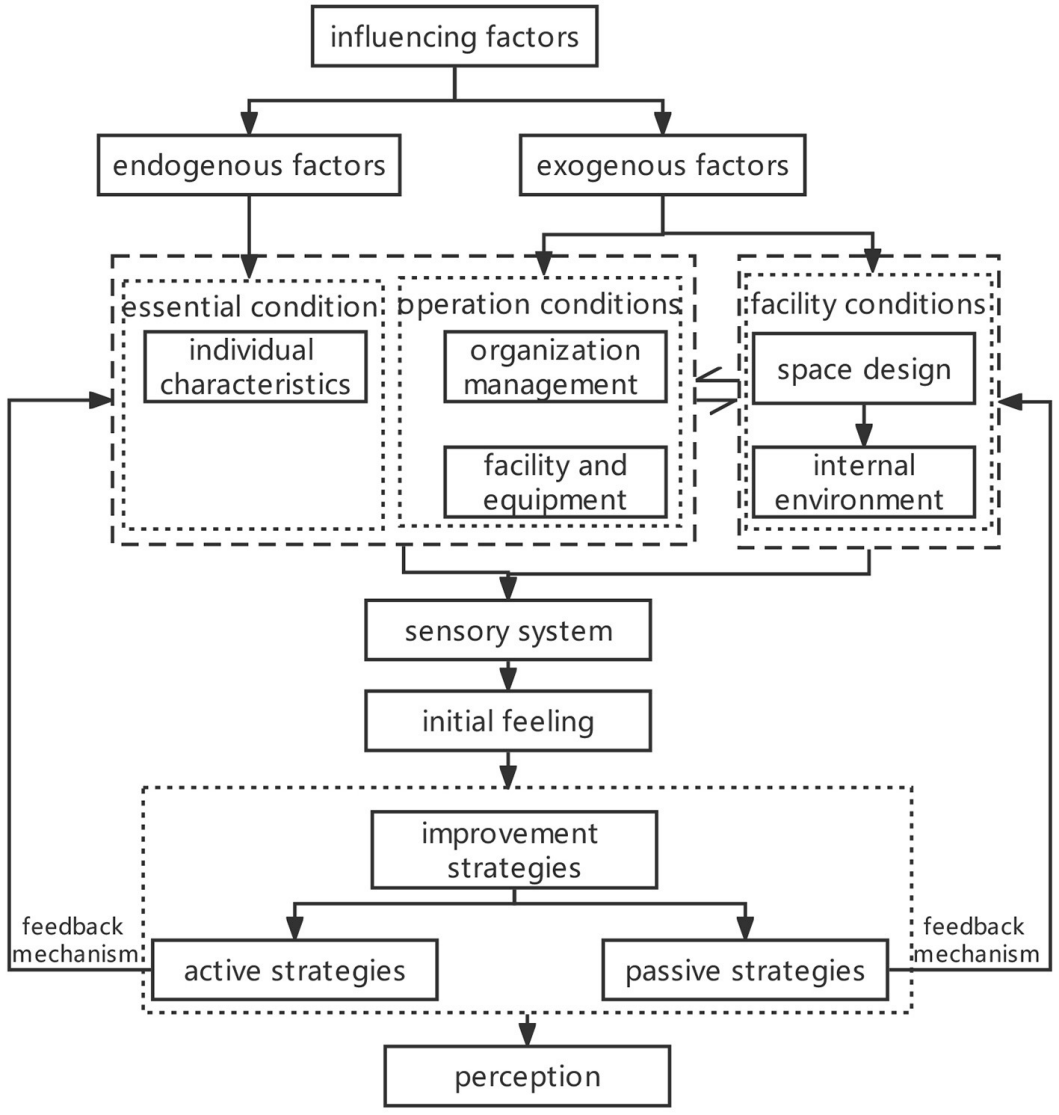
Perception models of healthcare workers in ACFs.

The relational structure of the perception model for the healthcare workers in ACFs is developed based on the interactions among the categories. According to this structure, the factors affecting the healthcare workers' perceptions can be further divided into two groups that are endogenous factors (individual characteristics) and exogenous factors (organization management, space design, internal environment and facilities and equipment). As endogenous factors are composed of individual characteristics, it is regarded as the basis of the workers' perceptions and the exogenous ones play rather critical roles. Both of them are merged together by the sensory system of the healthcare workers and then the primary perception is produced. The improvement measures that counter healthcare workers' adverse perceptions can be further classified into two parts: active and passive strategies (Figure 3).

## Discussion

The passive design strategy improves the performance of the building through the optimization of the building design, like the appearance and space design of the building and the selection of building materials. The active strategy aims to enhance building performance by artificial supplementary measures, such as air-conditioners and the operations of the buildings. The design of a hospital is usually improved by analyzing the workflow and needs of healthcare workers, by which the designer can ensure better user perception for the healthcare workers *via* promoting the design of space and environment of the buildings. ACFs are some of the most promising solutions to the pressing health care needs under disaster situations. During the COVID-19 epidemic, previous studies show that the environment of the ACFs not only has adverse impacts on the patients but requires healthcare workers to adjust themselves to the new surroundings (59). To improve healthcare workers' perceptions of ACFs during the epidemic, administrators of ACFs focus on active measures by a multi-tiered care model, PPE packs, emergency medical staff training, and psychological crisis intervention (49, 60–62). Recently, there have been studies on passive measures concerning more about the safety of the buildings' functional layout and internal environment (63–66). Although security is foremost in the ACFs, it is also essential to consider the healthcare workers' perceptions, which may reduce operating costs and active remedial measures. However, previous studies seldom explained the contributing factors of ACFs' design affecting the perception of healthcare workers. Only some showed that buildings, like residential living situations, impacted people's physical and mental well-being during the epidemic (67–69). Some analyzed the effect of housing built-environments on personal depression and anxieties (70, 71). Also, studies using multiple regression analyses show that the better the building design is, the fewer stress people may feel and the more active feedback the user will get (72). Passive design measures, such as function division, interior design, socialization approaches to design and positive distraction of light and sound can improve people's behaviors and emotions, reduce pressure and anxiety, and enhance users' perception and satisfaction (73).

Because healthcare environments are one of the most complex and demanding fields of work, an interdisciplinary solution is needed to achieve the goal of passively improving the healthcare environment. Giuliano K. K. and other researchers proposed that a nurse-engineer partnership is one of the most promising solutions to health care issues. Although the nurse-engineer partnership is faced with many barriers, it is encouraging to empower both nurses and engineers to create collaborations. According to Giuliano, finding a way for engineers to be trained in nursing and nurses to enter engineering are strategies helpful to developing infrastructure for health care innovation (74). For example, Brambilla and other researchers proposed the massive vaccination center layouts with the passive strategies, which is not only address safety by reducing cross-contamination risks, and improve the process efficiency but also ensure healthcare workers' well-being by the designs of resting spaces, short distances, and the correct sizing of space for the different activities (75). Meanwhile, they developed an easy-to-use checklist divided into two sections containing general and specific structural requirements to ensure the different activities' quality, safety, and efficiency (76). In addition, relevant researches also show that it is necessary to strengthen the synergy between design and health and training multidisciplinary professionals for future healthcare (77, 78).

The above discussions show that building characteristics affect personal perceptions during the pandemic, and the optimization of the built facilities can improve healthcare workers' health and well-being. Therefore, it is necessary to strengthen the emerging multidisciplinary education, which can develop the nurse-engineer partnership, to excavate passive improvement strategies for seeking more optimization measures for the building design of the ACFs. Specifically, such passive measures include the number of beds in each care unit in the bed area, the layout of healthcare workers passage and patient passage, the openness and accessibility of nurse stations, and the position of medical apparatus and instruments. The optimization of the building design and environment of the ACFs can be realized by the passive strategies, reducing the healthcare workers' adverse perceptions and the operating costs and active measures.

## Conclusion

The research aims to analyze the contributing factors to the healthcare workers' perceptions of the ACFs in this pandemic. Analyzing the actual narration of healthcare workers can avoid presupposition by the researchers through a qualitative study based on Grounded Theory. Eventually, there are five factors affecting the healthcare workers' perceptions which can be further divided into endogenous factors and exogenous factors. By interpreting the interactions among the factors and perception of healthcare workers, the passive strategies are realized to protect people's health and well-being in ACFs. In all, the research broadens new horizons for future research on the optimization of ACFs. It is also suggested that the emerging multidisciplinary education should be strengthened, especially the nurse-engineer partnership. Furthermore, exploring the measures of the rebuilding facilities as many as possible can help improve healthcare workers' perceptions and protect the health and well-being of people in ACFs.

## Limitations

Although this paper proposed a way to optimize the healthcare workers' perception of ACFs based on passive design, it did not explore specific measures which need further research. In addition, the healthcare workers interviewed are all from China. As mentioned above, the ACFs have played various roles in different countries during the epidemic (14–16), which leads to the differences in the responsibilities and working environment of the healthcare workers. Meanwhile, the interviewees come from other provinces to fight the epidemic in Wuhan, which means that their adverse perceptions may not be influenced by the fear that their families could be infected by the virus, as shown by some studies (44). Limitations as such may constrain the feasibility of this research and lead to differences in some details of the factors of perception in ACFs in different regions.

## Data availability statement

The raw data supporting the conclusions of this article will be made available by the authors, without undue reservation.

## Ethics statement

Ethical review and approval was not required for the study on human participants in accordance with the local legislation and institutional requirements. Written informed consent for participation was not required for this study in accordance with the national legislation and the institutional requirements.

## Author contributions

HW is responsible for interview data collection, article writing, and post revision. PL is responsible for interview design and article content inspection. YW is responsible for interview data collection and post revision. XZ is responsible for post revision. All authors contributed to the article and approved the submitted version.

## Funding

This research was funded by the National Natural Science Foundation of China, grant number 52078156.

## Conflict of interest

The authors declare that the research was conducted in the absence of any commercial or financial relationships that could be construed as a potential conflict of interest.

## Publisher's note

All claims expressed in this article are solely those of the authors and do not necessarily represent those of their affiliated organizations, or those of the publisher, the editors and the reviewers. Any product that may be evaluated in this article, or claim that may be made by its manufacturer, is not guaranteed or endorsed by the publisher.
